# The distribution and spectrum of thalassemia variants in GUIYANG region, southern China

**DOI:** 10.1186/s13023-025-03569-8

**Published:** 2025-02-07

**Authors:** Xuanyin Zhao, Zhiyu You, Yunyan Deng, Yi Zhou, Dongyang Deng, Jian Quan, Fang Chen, Zhimei Yan, Ya Qi, Leilei Chen, Fang Xiang, Weixian Zheng, Ruyi Zhang

**Affiliations:** 1 Department of Obstetrics, First Affiliated Hospital of Guizhou University of Traditional Chinese Medicine, Guizhou, People’s Republic of China; 2 Department of Clinical Laboratory, First Affiliated Hospital of Guizhou University of Traditional Chinese Medicine, Guizhou, People’s Republic of China; 3 Department of Clinical Laboratory, Anshun Hospital of Guizhou Aviation Industry Group, Guizhou, People’s Republic of China; 4 Graduate School, Guizhou University of Traditional Chinese Medicine, Guizhou, People’s Republic of China; 5 Center for Eugenics Research, First Affiliated Hospital of Guizhou University of Traditional Chinese Medicine, No.71 Baoshan North Road, Yunyan District, Guiyang City, 550001 People’s Republic of China

**Keywords:** Thalassemia, Guizhou Province, Molecular characterization

## Abstract

Thalassemia is one of southern China’s most common inherited disorders. This retrospective study analyzed the results of thalassemia gene testing conducted on 20,478 individuals from January 1, 2019, to April 31, 2024 in the First Affiliated Hospital of Guizhou University of Traditional Chinese Medicine. The cohort consisted of 19,733 females and 745 males, with 1401 individuals testing positive for thalassemia. Among the positive cases, 942 had α thalassemia, 431 had β thalassemia, and 25 had variants in both α and β thalassemia genes. Interestingly, a subgroup of individuals with thalassemia variants not previously documented in medical literature was identified. The study highlighted the prevalence of thalassemia among different ethnic groups, with individuals of Han ethnicity being the most affected. Geographical analysis revealed a concentration of cases in Guizhou Province, particularly in Guiyang city, Bijie, and Qiannan Prefecture. These findings provide valuable insights into the epidemiology of thalassemia in the region and the distribution of affected individuals.

**Supplementary Information:**

The online version contains supplementary material available at 10.1186/s13023-025-03569-8.

## Introduction

Thalassemia is a significant autosomal recessive blood disorder characterized by the disrupted synthesis of one or more globin proteins, which leads to alterations in both the structure and function of hemoglobin [1, 2]. This malformation compromises the oxygen-carrying capacity of erythrocytes, initiating varying degrees of hemolytic anemia [3]. The disorder predominantly manifests in warmer equatorial regions, with a notably high prevalence in the southern provinces of China, where specific genetic variations are more common [4]. Globally, it is estimated that approximately 400 million individuals are carriers of thalassemic variants, affecting populations in over 160 countries [5]. Thalassemia is categorized into three main forms: alpha (α-), beta (β-), and delta (δ-) thalassemia. Interestingly, a subgroup is distinguished by the specific globin gene variant involved [6].

Of these, α- and β-thalassemia are the most prevalent; α-thalassemia generally arises from gene deletions, while β-thalassemia is predominantly caused by point mutation [7].

The clinical manifestations of thalassemia can vary significantly, ranging from mild and asymptomatic cases to severe, life-threatening conditions, depending on the specific location and type of thalassemia gene variants. The synthesis of α chains is regulated by four genes (αα/αα), and the severity of clinical and analytical abnormalities depends on whether one, two, three, or all four α genes are absent. The deletion of a single gene is typically asymptomatic, whereas the deletion of all four genes can lead to hydrops fetalis, which is incompatible with life. In contrast, β chain synthesis is regulated by only two genes (β/β), resulting in a narrower range of clinical consequences: from asymptomatic carriers (β/0) to severe forms of hemolytic anemia, depending on the specific DNA variant affecting both β genes [8]. Individuals with thalassemia minor may show little to no signs of anemia, whereas severe forms of α-thalassemia can result in fetal or neonatal mortality, sometimes causing complications for the mother as well [9]. In contrast, children with β-thalassemia major develop pronounced anemia soon after birth, requiring lifelong management strategies, including regular blood transfusions and chelation therapy to manage iron overload [10].

Physically, significant cases of thalassemia are characterized by symptoms such as fatigue, weakness, shortness of breath, pallor, and splenomegaly among other complications [2, 4, 11]. The broader impact of thalassemia on families and communities highlights the necessity of robust public health strategies such as carrier screening and genetic counseling, aimed at reducing its incidence [12]. However, these public health measures are supported by significant advancements in molecular diagnostics, which have enhanced our understanding of the genetic underpinnings of thalassemia. Recent research developments have led to the identification of rare variants, furthering our knowledge and enabling targeted interventions [12].

Building on these insights, we have compiled and analyzed thalassemia detection data from Guiyang region, capital of Guizhou Province in South China. This analysis not only contributes to our understanding of regional genetic variability but also underscores the ongoing necessity for comprehensive public health initiatives and improved educational programs aimed at managing and reducing the burden of thalassemia globally.

## Materials and methods

### Population

This study was conducted at the First Affiliated Hospital of Guizhou University of Traditional Chinese Medicine where a total of 20,478 samples were collected for thalassemia gene screening from January 1, 2019, to March 31, 2024. Samples were obtained from routine prenatal screenings, physical examinations, and inpatient evaluations tailored for individuals suspected of having thalassemia. Additionally, samples displaying phenotypic traits suggestive of thalassemia, yet undiagnosed via the commercial diagnostic kit from Hybribio Company, were collected. Alternative diagnostic methods, such as electrophoresis and sequencing, were utilized to confirm the presence of thalassemia variants. This multifaceted diagnostic approach enabled a more comprehensive identification of thalassemic variants and improved the diagnostic accuracy through the integration of advanced molecular techniques, thereby enriching the understanding of thalassemia’s genetic epidemiology in the region.

### Reagents and instruments

The reagents used included the Hybribio Blood Genomic DNA Extraction Kit and the Mediterranean Anemia Hybridization Reagent. The principal instrument employed was the HB2012A Nucleic Acid Molecular Hybridization Instrument from Guangdong Hybribio Biotech Company. For sample collection, 2 ml of venous blood was drawn from each participant into an EDTA anticoagulant tube and stored at 2–8 °C.

### Flow-through hybridization and gene chip (FHGC)

The PCR mix was retrieved from the − 20 °C freezer 30 min beforehand and thawed at 4 °C before centrifugation. Similarly, the Taq enzyme, also stored at − 20 °C, was briefly centrifuged following retrieval. Both the PCR mix and the enzyme, after centrifugation, had 5 μl of DNA solution added, mixed, and centrifuged once more before being placed in the PCR machine for amplification. The PCR cycle parameters were set as follows: initial denaturation at 95 °C for 15 min, followed by 35 cycles of 97 °C for 50 s, 60 °C for 60 s, 72 °C for 120 s, with a final extension at 72 °C for 19 min. PCR products were stored at 4 °C after amplification.

For hybridization, PCR products were heated in the PCR machine at 95 °C for 10 min for denaturation and quickly cooled in an ice bath. The hybridization instrument was set up by placing the metal plate, separation membrane, and adding distilled water, followed by draining the water completely with the pump. Next, the hybridization membrane was arranged, topped with silicone rings and the separation chamber, and the cover snapped on. After draining the water completely using the pump, 0.8 ml of pre-warmed hybridization solution was added for pre-hybridization at 42 °C. This was followed by adding the denatured α-thalassemia and β-thalassemia PCR products for capillary hybridization with fresh hybridization solution for 30 min at 42 °C. Washing the hybridization membrane involved the use of warmed solution WB1, followed by a temperature reset to 25 °C for blocking and enzyme labeling, with subsequent washes and development steps until the final washing with distilled water and drying between absorbent paper sheets. Results were interpreted as per manual instructions, with ambiguous results sent to Guangdong Hybribio Biotech for sequencing verification.

### Statistical analysis

Continuous variables were summarized using descriptive statistics, including means and ranges or standard deviations, while categorical variables were presented as counts and percentages. All statistical analyses were conducted using Microsoft office Excel and R software.

## Results

### Characteristics of the studied population

Between January 1, 2019, and March 31, 2024, our center conducted a thalassemia gene testing on a total of 20,478 individuals. As most samples come from pregnant women during prenatal screening, the study population consisted of 19,733 females and 745 males. The cohort included 19,928 individuals who were undergoing routine medical examinations and 550 hospitalized patients. Age distribution within the study sample revealed that 155 individuals were younger than 1 year, 686 were aged between 1 and 22 years, and 19,637 were older than 22 years.

**Fig. 1:**
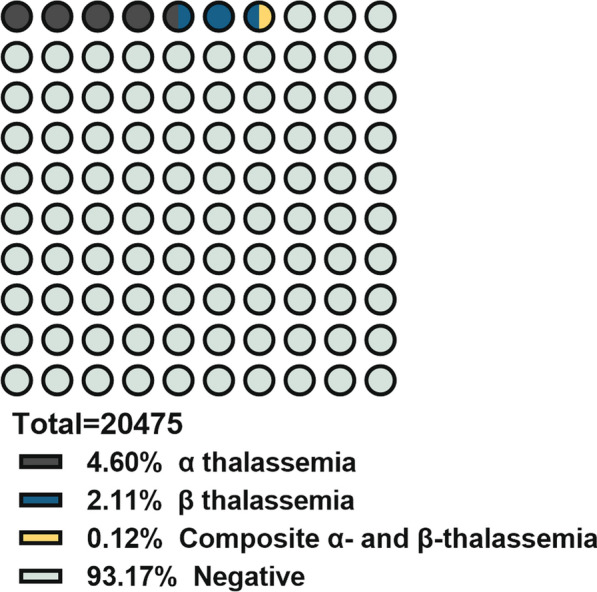
Composition of study population included

In this retrospective analysis, it was found that 1401 individuals tested positive for the presence of the thalassemia gene, representing a positive rate of 6.84%. This group was predominantly female, with 1196 individuals testing positive, compared to 205 males. The positive cases were categorized as 1167 individuals who were part of the routine medical examination group and 234 who were hospitalized patients. The age distribution of positive cases indicated that 27 individuals were younger than 1 year, 113 were aged between 1 and 22 years, and 1261 were older than 22 years (Table 1 and Fig. 1).

**Table 1:** Characteristics of the client population

	All of subjects (Number of people)	Thalassemia positive (Number of people) (%)
Female	19,733	1196 (6.06)
Male	745	205 (27.52)
Health examination	19,928	1167 (5.86)
Hospitalization	550	234 (42.55)
Age (years)		
< 1	155	27 (17.42)
> 1, ≤ 22	686	113 (16.47)
≥ 22	19,637	1261 (64.22)

### Prevalence of thalassemia

Of the 1401 individuals identified as positive for thalassemia, a detailed analysis revealed that 942 individuals harbored α thalassemia, 431 individuals presented with β thalassemia, and 25 individuals manifested variants involving both α and β thalassemia genes. Among those with α thalassemia, the most prevalent variant was the 3.7 heterozygous variant, with subsequent occurrences of SEA deletion, α4.2 deletion, and α^CS^ variant. In the case of β thalassemia, the CD17 heterozygous variant emerged as the most common, followed by CD41-42, IVS-II-654, and βEM variant.

Of particular interest, a subgroup of seventeen individuals displayed thalassemia variants that had not been previously documented in medical literature. Within this subgroup, 5 individuals exhibited α thalassemia variant, 9 individuals displayed β thalassemia variant, and 3 individuals presented with variant involving both forms of thalassemia (Tables 2, 3, 4 and 5, supplement Tables 1 and 2).

**Table 2:** Distribution of α-thalassemia genotypes

Genotype	Counts (n)	Percentages in α-thalassemia	Hb (g/L) (mean ± SD)	MCV (fL) (mean ± SD)	MCH (pg/cell) (mean ± SD)
-α^3.7^/deletion	459	48.99%	123.06 ± 15.59	85.28 ± 6.47	28.14 ± 2.46
_SEA/deletion	226	24.12%	111.60 ± 19.44	70.52 ± 6.36	22.49 ± 2.36
-α^4.2^/deletion	89	9.50%	125.04 ± 14.89	86.27 ± 5.78	28.39 ± 2.05
-α^CS^/mutation	82	8.75%	110.95 ± 23.24	83.12 ± 7.76	26.84 ± 3.25
-α^WS^/mutation	23	2.45%	121.32 ± 14.91	83.60 ± 6.52	27.60 ± 2.67
-α^QS^/mutation	13	1.39%	110.85 ± 18.85	77.27 ± 4.62	24.51 ± 2.73
αααanti4.2	6	0.64%	100.00 ± 17.28	73.30 ± 6.86	23.10 ± 2.56
HBA2:c.300 + 55T > G	5	0.53%	119.10 ± 14.42	69.44 ± 9.35	22.02 ± 4.41
_SEA/-α^QS^	4	0.43%	72.00 ± NA	68.00 ± NA	17.90 ± NA
HBA1:c.300 + 55G > T	4	0.32%	121.00 ± 12.42	91.63 ± 1.60	29.80 ± 1.56
_SEA/-α^CS^	3	0.32%	73.33 ± 30.73	67.20 ± 4.22	19.77 ± 1.33
THAI	3	0.32%	119.50 ± 10.61	70.35 ± 1.74	22.80 ± 1.76
-α^3.7^/_SEA	2	0.21%	68.00 ± 3.00	60.10 ± 7.40	17.40 ± 1.40
-α3.7/-α3.7	2	0.21%	112.5 ± 0.71	75.00 ± 0.85	24.20 ± 0
-α^3.7^/-α^CS^	2	0.21%	126.00 ± 6.00	73.70 ± 3.90	26.45 ± 4.35
-α4.2/HBA1:c.223G > C	1	0.11%	143 ± NA	78.9 ± NA	26.3 ± NA
HBA1:c.95 + 1G > A	1	0.11%	150.00 ± NA	80.40 ± NA	25.60 ± NA
_SEA/HBA2:c.427T > C	1	0.11%	74.00 ± NA	74.90 ± NA	19.00 ± NA
HBA1:c.104T > G	1	0.11%	123.00 ± NA	86.70 ± NA	29.60 ± NA
HBA1:c.273G > C	1	0.11%	124.20 ± NA	91.10 ± NA	31.50 ± NA
HBA1:c.84G > T	1	0.11%	127.00 ± NA	79.80 ± NA	25.30 ± NA
HBA2:c.159T > G	1	0.11%	127.00 ± NA	84.40 ± NA	25.50 ± NA
HBA2:c.218A > G	1	0.11%	135.00 ± NA	76.50 ± NA	24.40 ± NA
HBA2:c.300 + 34G > A	1	0.11%	116.00 ± NA	84.30 ± NA	26.70 ± NA
HBA2:c.377T > C	1	0.11%	^#^132.00 ± 1.80	^#^75.20 ± 3.30	^#^24.5 ± 0.50
-α^QS^/αααanti3.7	1	0.11%	95.00 ± NA	79.40 ± NA	22.40 ± NA
HBA2:c.51G > T	1	0.11%	155.00 ± 28.28	92.60 ± 1.13	32.50 ± 0.85
HBA2:c.80C > A	1	0.11%	124.00 ± NA	85.70 ± NA	30.00 ± NA
αααanti3.7	1	0.11%	99.00 ± NA	84.20 ± NA	26.90 ± NA
Total	937	100	NA	NA	NA

^#^Data are derived from other literature or databases

**Table 3:** Distribution of β-thalassemia genotypes

Genotype	Counts (n)	Percentages in β-thalassemia (%)	Hb (g/L) (mean ± SD)	MCV (fL) (mean ± SD)	MCH (pg/cell) (mean ± SD)
CD17	154	36.58	104.48 ± 19.86	68.83 ± 9.16	21.95 ± 3.49
CD41-42	125	29.69	104.04 ± 17.64	69.13 ± 9.54	22.12 ± 3.56
IVS-II-654	60	14.25	96.81 ± 18.70	69.39 ± 7.27	21.88 ± 2.55
βEM	15	3.56	118.37 ± 11.81	83.60 ± 4.51	27.75 ± 1.31
CapM	11	2.61	129.00 ± 10.26	92.12 ± 4.37	30.99 ± 1.45
CD43	8	1.90	93.27 ± 20.56	71.41 ± 6.08	22.18 ± 1.54
HBB:c.341T > A	8	1.90	127.20 ± 17.65	87.33 ± 3.39	28.57 ± 1.21
-28C/N	6	1.43	128.86 ± 7.54	72.80 ± 4.95	23.87 ± 1.25
CD71-72	5	1.19	90.92 ± 14.22	65.75 ± 4.81	20.77 ± 1.47
CD27-28	4	0.95	110.67 ± 10.97	67.23 ± 1.27	22.20 ± 0.87
HBB:c.170G > A	3	0.71	125.50 ± 7.50	93.07 ± 3.86	31.50 ± 2.13
HBB:c.162delT	2	0.48	111 ± NA	58.1 ± NA	18.8 ± NA
HBB:c.180G > C	2	0.48	121.20 ± 7.40	92.75 ± 1.25	30.35 ± 1.05
HBB:c.316-179A > C	2	0.48	101.50 ± 8.50	100.25 ± 21.35	31.80 ± 7.80
-29C/N	1	0.24	96.00 ± NA	76.10 ± NA	26.40 ± NA
HBB:c.341T > A	1	0.24	161 ± NA	90.3 ± NA	30.6 ± NA
HBB:c.170G > A	1	0.24	124 ± NA	97.2 ± NA	31.2 ± NA
HBB:c.100G > A	1	0.24	^#^122.00 ± NA	^#^80.60 ± NA	^#^26.4 ± NA
HBB:c.113G > A	1	0.24	113 ± NA	66.1 ± NA	20.8 ± NA
HBB:c.130G > C	1	0.24	127 ± NA	83.2 ± NA	27.8 ± NA
HBB:c.246C > A	1	0.24	122 ± NA	78.4 ± NA	25.1 ± NA
HBB:c.27G > C	1	0.24	^#^124.80 ± 0.79	^#^94.78 ± 10.96	^#^30.33 ± 3.7
HBB:c.304G > C	1	0.24	^#^105.00 ± NA	^#^82.70 ± NA	^#^26.00 ± NA
HBB:c.327C > T	1	0.24	115 ± NA	75.9 ± NA	23.5 ± NA
HBB:c.364G > C	1	0.24	114 ± NA	108.2 ± NA	36.7 ± NA
HBB:c.68A > C	1	0.24	156 ± NA	84.4 ± NA	29 ± NA
βEM / CD27-28	1	0.24	94.20 ± NA	63.10 ± NA	20.30 ± NA
HBB:c.92 + 2T > C	1	0.24	^#^96.00 ± NA	^#^55.7 ± NA	^#^17.8 ± NA
HBB:c.315 + 5G > C	1	0.24	^#^94.5 ± NA	^#^48.99 ± NA	^#^15.81 ± NA
HBB:c.316-146T > G	1	0.24	^#^121.00 ± NA	^#^59.60 ± NA	^#^18.60 ± NA
Total	421	100	NA	NA	NA

^#^Data are derived from other literature or databases

**Table 4:** Distribution of composite α- and β-thalassemia genotypes

Genotype		Counts (n)	Percentages in α-composite β-thalassemia (%)	Hb (g/L) (mean ± SD)	MCV (fL) (mean ± SD)	MCH (pg/cell)
α	β					
-α3.7	CD41-42	4	16.00	115.00 ± 20	67.00 ± 1.80	22.05 ± 1.25
-α3.7	CD17	3	12.00	86.00 ± NA	65.10 ± NA	20.60 ± NA
_SEA	CD41-42	2	8.00	NA	NA	NA
_SEA	CD17	2	8.00	NA	NA	NA
-α3.7	IVS-II-654	2	8.00	126.00 ± NA	92.90 ± NA	32.00 ± NA
-α3.7	βEM	1	4.00	157 ± NA	84.6 ± NA	28.8 ± NA
_SEA	IVS-II-654	1	4.00	138 ± NA	67.9 ± NA	22 ± NA
-αCS	IVS-II-654	1	4.00	113 ± NA	67.6 ± NA	20.7 ± NA
_SEA	CD27-28	1	4.00	117.00 ± NA	66.50 ± NA	22.70 ± NA
-αCS	CD41-42	1	4.00	109.00 ± NA	67.70 ± NA	21.10 ± NA
_SEA	CD14-15	1	4.00	NA	NA	NA
-αCS	βEM	1	4.00	NA	NA	NA
-α4.2	CD17	1	4.00	NA	NA	NA
_SEA	HKαα	1	4.00	NA	NA	NA
HBA2:c.-24C > G	HBB:c.315 + 180T > C	1	4.00	116 ± NA	78 ± NA	25.3 ± NA
HBA2:c.300 + 55T > G	HBB:c.34G > A	1	4.00	97 ± NA	8 1.50 ± NA	24 ± NA
HBA2:c.300 + 55T > G	HBB:c.315 + 252C > T	1	4.00	NA	73.3 ± NA	23 ± NA
Total		25	100	NA	NA	NA

**Table 5:** Genotypes of unreported thalassemia

HGVS nomenclature	Mutation type	Hb (g/L)	MCV (fL)	MCH (pg/cell)
HBA1:c.300 + 35G > A	α-thalassemia	NA	105	37.4
HBA2:c.193C > T	α-thalassemia	NA	87.3	31.4
HBA2:c.316C > T	α-thalassemia	NA	73.8	23.8
NC_000016.10:g.165401_184701del/ NC_000016.10:g.169818_174075del/HBA1:c.223G > C	α-thalassemia	35	86.8	24.3
HBB:c.315 + 300-308AAAAAAAAA > AAAAAAAA	α-thalassemia	80	122.4	39.8
HBB:c.-153C > A	β-thalassemia	NA	66.5	20
HBB:c.*34G > A	β-thalassemia	NA	74.1	22.4
HBB:c.-107A > C	β-thalassemia	NA	80.4	22.4
HBB:c.-23 A > G	β-thalassemia	108	78.3	24.1
HBB:c.315 + 299–308 AAAAAAAAA > AAAAAAAA	β-thalassemia	NA	76.8	23.8
HBB:c.315 + 300-308AAAAAAAAA > AAAAAAAAAA	β-thalassemia	115.00 ± 5.00	81.30 ± 9.60	25.85 ± 5.25
HBB:c.316–113 A > T	β-thalassemia	82	65.7	17.6
HBB:c.316–31 C > T	β-thalassemia	110	77	25.6
HBB:c.315 + 300-308AAAAAAAAA > AAAAAAAA	β-thalassemia	NA	58.4	13.5
HBA1:c.196 G > A /HBB:c.-11-8delAAAC	α-composite β-thalassemia	NA	NA	NA
HBA1:c.196G > A/HBB:c.52A > T	α-composite β-thalassemia	122	60.3	19.7
NG_000006.1:g.34164_37967del3804/HBA2:c.427 T > C/HBB:c.52A > T	α-composite β-thalassemia	NA	NA	NA

Guizhou is home to many ethnic minorities, and a significant number of unique ethnic groups reside in the area, some of whom may carry distinctive thalassemia variant. Our retrospective analysis indicated that among those who tested positive for thalassemia, individuals of Han ethnicity constituted the highest proportion, followed by those from Buyi, Miao, and Tujia ethnic groups (Table 6).

**Table 6:** The ethnic distribution of thalassemia in Guizhou

Ethnic	α-thalassemia (%)	β-thalassemia (%)	Total composition (%)
HAN	725 (82.48)	335 (93.31)	1060 (85.62)
BUYI	18 (2.05)	10 (2.79)	28 (2.26)
MIAO	12 (1.37)	6 (1.67)	18 (1.45)
TUJIA	3 (0.34)	3 (0.84)	6 (0.48)
YI	3 (0.34)	1 (0.28)	4 (0.32)
DONG	2 (0.23)	1 (0.28)	3 (0.24)
QILAO	1 (0.11)	2 (0.56)	3 (0.24)
SHUI	1 (0.11)	1 (0.28)	2 (0.16)
BAI	1(0.11)	0 (NA)	1 (0.08)
Total	879 (100)	359 (100)	1238 (100)

### Geographical distribution of the thalassemia population

**Fig. 2:**
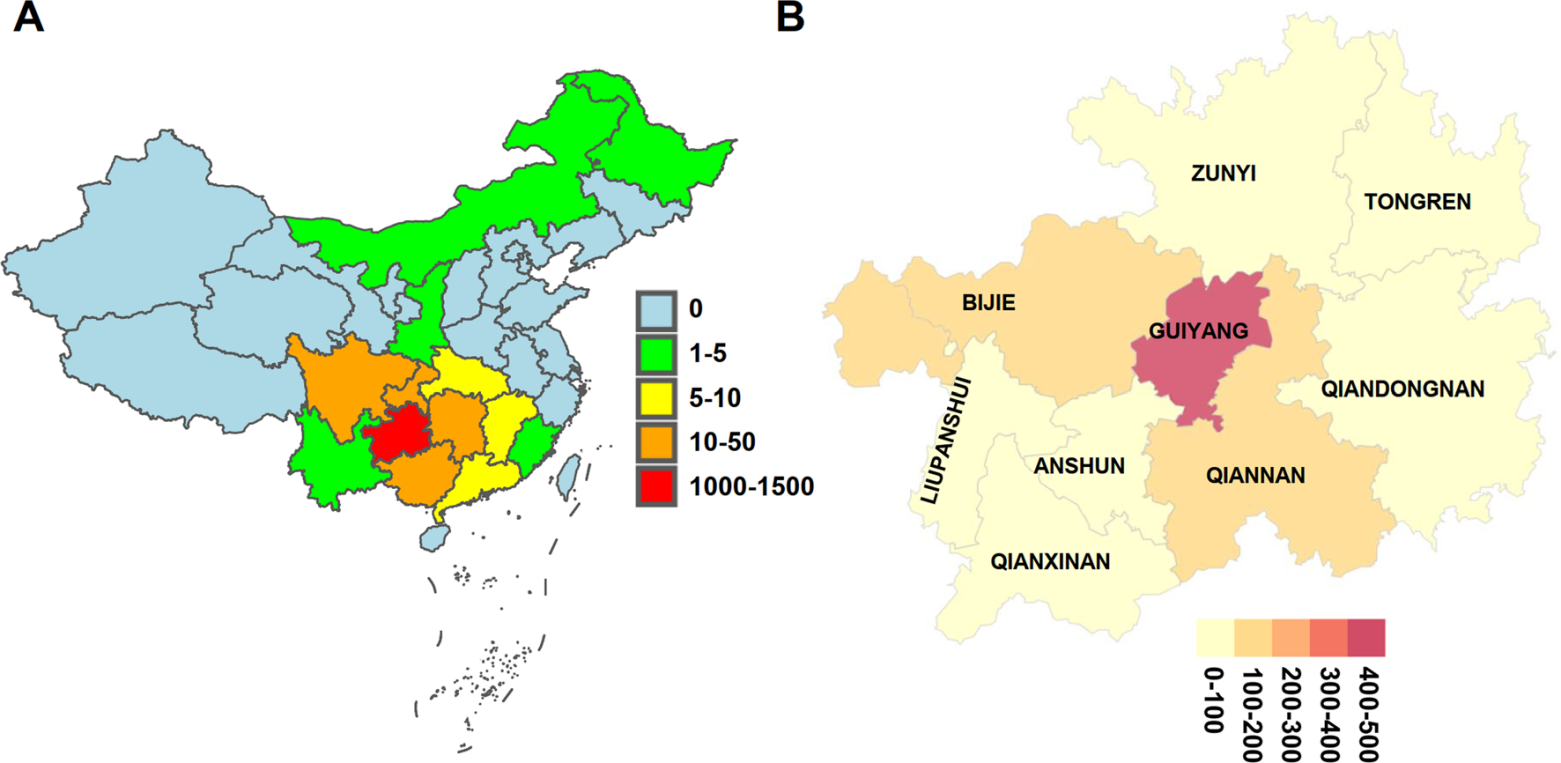
The distribution of the positive population included in the research on the map

To delve deeper into the spatial distribution of thalassemia carriers, we conducted an analysis whereby the household registration locations of affected individuals were utilized to generate a population distribution map. This geographical representation unveiled that the majority of individuals testing positive for thalassemia gene variants within our study cohort were predominantly concentrated in Guizhou Province, with additional clusters observed in adjacent provinces. Within the delineated 9 major administrative regions of Guizhou Province, it was discerned that the highest density of affected individuals was discerned in Guiyang city, with subsequent concentrations noted in Bijie and Qiannan Prefecture (Fig. 2).

## Discussion

Thalassemia is a group of inheritable single-gene disorders that pose a significant global public health concern due to their potential to result in fetal deaths or birth defects [13]. The two most common types of thalassemia are α- and β-thalassemia [14]. In α-thalassemia, genetic variants typically entails large deletions in the α-globin gene, with a smaller proportion caused by point mutation. Conversely, β-thalassemia is predominantly caused by point mutation in the β-globin gene. While most individuals with thalassemia are asymptomatic carriers of the thalassemia deletion gene, a minority present with microcytic hypochromic anemia. The primary treatments for severe manifestations of thalassemia include blood transfusions and iron chelation therapy, both of which are palliative measures that do not address the underlying cause of the disorder [15]. Consequently, patients with thalassemia require lifelong, continuous treatment, placing a substantial financial burden on families and society. Although promising gene therapies for thalassemia have emerged, their long-term safety and efficacy remain to be determined. The primary focus of thalassemia management involves increasing awareness and preventive measures [16]. By promoting education about the risks of thalassemia and encouraging widespread thalassemia screening, particularly among prospective parents, the incidence of moderate to severe forms of the disorder can be effectively reduced. In the Guiyang area, all pregnant women are screened for thalassemia in the first trimester. Some institutions perform hemoglobin electrophoresis screening, and if abnormal hemoglobin is detected, they proceed with genetic screening. Other institutions directly screen for thalassemia genes. If a pregnant woman is found to be a carrier of the thalassemia gene, her husband may also be screened upon a doctor's recommendation. If the husband is also found to carry the thalassemia gene, some pregnant women may receive a prenatal diagnosis after a doctor's assessment to check for the risk of severe thalassemia in fetus.

Currently, the detection of thalassemia primarily involves the utilization of FHGC, high-resolution melting analysis (HRMA), next-generation high-throughput sequencing, third-generation sequencing, gel electrophoresis, first-generation sequencing, and multiplex ligation-dependent probe amplification (MLPA) alongside validation [17, 18]. FHGC and HRMA are favored for their simplicity and cost-effectiveness in concurrently identifying multiple common mutation sites, rendering them essential tools in early thalassemia genetic screening. The advancement and enhancement of technology have propelled next-generation sequencing to the forefront in numerous clinical settings, improving throughput and encompassing a broader spectrum of mutation sites [19].

Third-generation sequencing, also referred to as long-read sequencing, signifies the latest breakthrough in DNA sequencing technology [20]. In contrast to traditional short-read sequencing approaches producing short DNA fragments typically measuring 100–300 base pairs, third-generation sequencing platforms can generate considerably longer reads, extending across thousands to tens of thousands of base pairs or even longer [21]. Although the utilization of third-generation sequencing can enhance the detection of rare variants sites, challenges such as high costs, the necessity for heightened precision, and extended reporting timelines currently limit its applicability in thalassemia genetic testing [22]. However, it is expected that as these obstacles are gradually overcome, third-generation sequencing will emerge as the predominant method for thalassemia gene analysis.

The approach employed for the identification of thalassemia gene mutations in this investigation incorporated FHGC in combination with agarose gel electrophoresis and first-generation sequencing for validation. Among the 20,478 subjects subjected to testing, 1401 individuals exhibited positive results for thalassemia gene mutations, yielding a positivity rate of 6.84%, which surpasses the national average carrier frequency of thalassemia standing at 3.62%. Within this cohort, 942 individuals were found to carry α-thalassemia variants, with a positivity rate of 4.60%, 431 individuals harbored β-thalassemia variants, reflecting a positivity rate of 2.11%, and 25 individuals presented with both α and β-thalassemia variants, presenting a positivity rate of 0.12%.

Comparative analysis of our findings with the documented carrier rates of 25% in individuals under the age of 18 and 3.28% in newborns within Guizhou Province reveals that our institution's positivity rate falls within this range [23, 24]. Discrepancies in positivity rates can be attributed to variances in the demographic composition of the study populations. Previous reports primarily focused on pediatric or neonatal populations, whereas our study cohort primarily consisted of healthy individuals undergoing routine medical assessments. This suggested that our data more accurately represent the prevalence of thalassemia carriers within the Guiyang region. Nevertheless, as our institution specializes in traditional Chinese medicine, the admission of patients with moderate to severe forms of thalassemia may be limited, potentially distorting the epidemiological profile of these patient groups. It is important to note that the majority of the population included in our study were women, as thalassemia screening is generally conducted among pregnant women, resulting in fewer men being involved. Additionally, 550 of the participants were hospitalized patients exhibiting symptoms related to thalassemia. These individuals, suspected of having thalassemia, underwent screening, which may cause the detection rate in this study to be slightly higher than the actual local detection rate. These problems may lead to statistical bias.

Through a retrospective examination, we identified 29 variations of α-thalassemia, 30 variants of β-thalassemia, 18 instances of combined α and β thalassemia, and 17 previously unreported variants. Contrary to previous assertions indicating _SEA as the predominant mutation in southern China [25], our investigation revealed that -α3.7 constituted the prevailing mutation (48.99%) within our subpopulation, followed by _SEA (24.12%) and -α4.2 heterozygosis (9.50%). Among the 28 identified β-thalassemia genotypes, the CD17 [codon 17 (A->T)] β0 heterozygote (36.58%), CD41-42 [41/42 (-TTCT)] β0 heterozygote (29.69%), and IVS-II-654 (C>T) β + heterozygote (14.25%) were the most prevalent variants, consistent with documented data from Guizhou Province but deviating from observations in Guangdong Province or Hainan Province [26, 27].

Furthermore, our study unveiled 17 novel mutation loci, 5 associated with α-thalassemia, 9 with β-thalassemia, and 3 with combined α and β thalassemia. Regrettably, due to constraints in data acquisition, comprehensive hematological parameters were only obtainable for carriers of 7 mutation sites, impeding the determination of the clinical significance of the remaining mutation loci.

The ethnically diverse landscape of Guizhou Province has fostered a unique spectrum of thalassemia variants. With the advent of modern transportation and increasing cultural integration, cohabitation and intermarriage among different ethnic groups have likely facilitated the spread of thalassemia carriers, including those with rare mutation sites. Our findings indicate that the majority of thalassemia carriers in the Guiyang region are from Han, Buyi, Miao, and Tujia ethnic backgrounds, reflecting their demographic prominence in the area. Given the ethnic diversity in Guizhou, we advocate for enhanced thalassemia screening to raise awareness about this disease.

Located in Guiyang, the capital of Guizhou Province, our center likely serves patients from across the province. By mapping thalassemia carriers based on their registered domiciles within Guizhou, we identified highest concentrations in Guiyang City, followed by Bijie City and Qiannan Prefecture. This is related to the baseline distribution of the population in the region. Additionally, thalassemia prevalence is notably higher in southern Chinese provinces like Guizhou, while significant carrier numbers are also observed in northern regions. However, consensus among experts regarding universal thalassemia screening in northern provinces remains lacking.

Advancements in thalassemia detection technology, from Sanger sequencing to next-generation methods, have significantly progressed. However, implementing large-scale screening remains economically challenging, considering regional disparities in China and variations in thalassemia epidemiology. Local health authorities must balance cost considerations with effective screening strategies tailored to local realities to minimize severe thalassemia cases.

## Conclusions

This study elucidated the presence of thalassemia carriers at our center through molecular characterization, while also considering ethnicity and regional dispersion. Our findings furnish valuable insights into the distribution of thalassemia within the Guiyang area, thereby aiding clinicians in assessing the likelihood of carriers transmitting severe thalassemia to offspring. Such insights hold utility in shaping policies aimed at thalassemia prevention and control. Future screening efforts should consider including northern Chinese populations in routine screening to prevent treatment delays and reduce births of children with severe thalassemia.

## Supplementary Information

Supplementary Material 1.

## Data Availability

The original contributions presented in the study are included in the article/Supplementary Material, further inquiries can be directed to the corresponding author. A data access agreement will need to be signed to gain access to the detailed data.

## Abbreviations

FHGC: Flow-through hybridization and gene chip
HRMA: High-resolution melting analysis
MLPA: Multiplex ligation-dependent probe amplification
