# Supplementary material for: The distribution and spectrum of thalassemia variants in GUIYANG region, southern China
Source: Orphanet J Rare Dis. 2025 Feb 7;20:56. doi: 10.1186/s13023-025-03569-8 (PMC11806605; doi:10.1186/s13023-025-03569-8)
Supplement: Supplementary file 1 — Supplementary Material 1. [file 13023_2025_3569_MOESM1_ESM.docx]

**sTable 1. Distribution of α-thalassemia genotypes.**

| **Genotype** | **Genotype Frequencies** | **Allele Frequencies** | **Genotype** | **Genotype Frequencies** | **Allele Frequencies** |
| --- | --- | --- | --- | --- | --- |
| - α^3.7^ / deletion | 2.24% | 0.513063 | _SEA / HBA2:c.427T>C | 0.005% | NA |
| _SEA / deletion | 1.10% | 0.267927 | HBA1:c.104T>G | 0.005% | 0.000556 |
| - α^4.2^ / deletion | 0.43% | 0.0995 | HBA1:c.273G>C | 0.005% | 0.000556 |
| - α^CS^ / mutation | 0.40% | 0.093385 | HBA1:c.84G>T | 0.005% | 0.000556 |
| - α^WS^ / mutation | 0.11% | 0.02557 | HBA2:c.159T>G | 0.005% | 0.000556 |
| - α^QS^ / mutation | 0.06% | 0.015564 | HBA2:c.218A>G | 0.005% | 0.000556 |
| αααanti4.2 | 0.03% | 0.00667 | HBA2:c.300+34G>A | 0.005% | 0.000556 |
| HBA2:c.300+55T>G | 0.02% | 0.007226 | HBA2:c.377T>C | 0.005% | 0.000556 |
| _SEA / - α^QS^ | 0.02% | NA | - α^QS^ / αααanti3.7 | 0.005% | NA |
| _SEA / - α^CS^ | 0.01% | NA | HBA2:c.51G>T | 0.005% | 0.000556 |
| THAI | 0.01% | 0.003335 | HBA2:c.80C>A | 0.005% | 0.000556 |
| HBA1:c.300+55G>T | 0.01% | 0.000556 | αααanti3.7 | 0.005% | 0.000556 |
| - α^3.7^ /_SEA | 0.01% | NA | HBA1:c.300+35G>A | 0.005% | 0.000556 |
| −α3.7 / −α3.7 | 0.01% | NA | HBB:c.315+300-308AAAAAAAAA>AAAAAAAA | 0.005% | 0.000556 |
| - α^3.7^ / - α^CS^ | 0.01% | NA | HBA2:c.193C>T | 0.005% | 0.000556 |
| - α4.2 /HBA1:c.223G>C | 0.005% | NA | HBA2:c.316C>T | 0.005% | 0.000556 |
| HBA1:c.95+1G>A | 0.005% | 0.000556 | NC_000016.10:g.165401_184701del / NC_000016.10:g.169818_174075del / HBA1:c.223G>C | 0.005% | NA |

Note: The complex heterozygous variation loci are not listed with allele frequencies.

**sTable 2. Distribution of β-thalassemia genotypes.**

| **Genotype** | **Genotype Frequencies** | **Allele Frequencies** | **Genotype** | **Genotype Frequencies** | **Allele Frequencies** |  |
| --- | --- | --- | --- | --- | --- | --- |
|  |  |  |  |  |  |  |
| CD17 | 0.75% | 0.379686 | HBB:c.246C>A | 0.01% | 0.00120919 |  |
| CD41-42 | 0.61% | 0.311971 | HBB:c.27G>C | 0.01% | 0.00120919 |  |
| IVS-Ⅱ-654 | 0.29% | 0.152358 | HBB:c.304G>C | 0.01% | 0.00120919 |  |
| βEM | 0.07% | 0.038694 | HBB:c.327C>T | 0.01% | 0.00120919 |  |
| CapM | 0.05% | 0.026602 | HBB:c.364G>C | 0.01% | 0.00120919 |  |
| CD43 | 0.04% | 0.019347 | HBB:c.68A>C | 0.01% | 0.00120919 |  |
| HBB:c.341T>A | 0.04% | 0.020556 | βEM / CD27-28 | 0.01% | NA |  |
| -28C/N | 0.03% | 0.01451 | HBB:c.92+2T>C | 0.01% | 0.00120919 |  |
| CD71-72 | 0.02% | 0.012092 | HBB:c.315+5G>C | 0.01% | 0.00120919 |  |
| CD27-28 | 0.02% | 0.012092 | HBB:c.316-146T>G | 0.01% | 0.00120919 |  |
| HBB:c.170G>A | 0.02% | 0.00120919 | HBB:c.315+300-308AAAAAAAAA>AAAAAAAA | 0.01% | 0.00120919 |  |
| HBB:c.162delT | 0.01% | 0.00120919 | HBB:c.-153C>A | 0.01% | 0.00120919 |  |
| HBB:c.180G>C | 0.01% | 0.00120919 | HBB:c.*34G>A | 0.01% | 0.00120919 |  |
| HBB:c.316-179A>C | 0.01% | 0.00120919 | HBB:c.-107A>C | 0.01% | 0.00120919 |  |
| -29C/N | 0.01% | 0.00120919 | HBB:c.-23 A>G | 0.01% | 0.00120919 |  |
| HBB:c.341T>A | 0.01% | 0.00120919 | HBB:c.315+299-308 AAAAAAAAA > AAAAAAAA | 0.01% | 0.00120919 |  |
| HBB:c.170G>A | 0.01% | 0.00120919 | HBB:c.315+300-308AAAAAAAAA>AAAAAAAAAA | 0.01% | 0.00120919 |  |
| HBB:c.100G>A | 0.01% | 0.00120919 | HBB:c.316-113 A>T | 0.01% | 0.00120919 |  |
| HBB:c.113G>A | 0.01% | 0.00120919 | HBB:c.316-31 C>T | 0.01% | 0.00120919 |  |
| HBB:c.130G>C | 0.01% | 0.00120919\ | HBB:c.315+300-308AAAAAAAAA>AAAAAAAA | 0.01% | 0.00120919 |  |

Note: The complex heterozygous variation loci are not listed with allele frequencies.
